# Applying Distinct Approaches to Racial and Ethnic Classification to the Surveillance of Obstetric and Neonatal Outcomes in the U.S. Military, 2010–2021

**Published:** 2026-02-26

**Authors:** Celeste J. Romano, Clinton Hall, Monica Burrell, Anna T. Bukowinski, Jackielyn Lanning, Sandra Maduforo, Sandra Michelle Magallon, Zeina G. Khodr, Gia R. Gumbs, Ava Marie S. Conlin

**Affiliations:** Deployment Health Research Department, Naval Health Research Center, San Diego, CA: Ms. Romano, Dr. Hall, Ms. Burrell, Ms. Bukowinski, Ms. Lanning, Ms. Maduforo, Ms. Magallon, Dr. Khodr, Ms. Gumbs, Dr. Conlin; Leidos, Inc., San Diego, CA: Ms. Romano, Dr. Hall, Ms. Burrell, Ms. Bukowinski, Ms. Lanning, Ms. Maduforo, Ms. Magallon, Dr. Khodr, Ms. Gumbs

## Abstract

Traditional, mutually exclusive approaches to racial and ethnic classification obscure important differences within major demographic groups and among multiracial populations. This study offers a novel examination of obstetric and neonatal outcomes among pregnant U.S. military service members, by applying multiple approaches to racial and ethnic classification and presenting disaggregated data. Overall, 235,608 births were identified among pregnant service members from 2010 through 2021. Inclusion of service members who identified with each racial group, whether alone or in combination with any other group, increased the American Indian or Alaska Native and Native Hawaiian or Pacific Islander birth populations by 209.7% and 94.0%, respectively, when compared to mutually exclusive classifications. Prevalences of obstetric outcomes such as cesarean delivery varied among racial and ethnic groups, particularly Asian and Latino populations, for example, Asian Indian, 36.7%; Filipino, 32.3%; Chinese, 26.5%; Puerto Rican, 30.2%; Mexican, 23.2%; and between distinct multiracial populations. Disaggregated estimates ultimately increased visibility of multiracial and Native service members and elucidated patterns indiscernible in aggregated data. Wider adoption of disaggregated racial and ethnic data methods is needed to improve understanding of health outcomes in the Military Health System.

**What are the new findings?**

Reporting of non-mutually exclusive racial and ethnic groups as well as disaggregated Asian, Hispanic or Latino, and multiracial populations elucidates important differences in obstetric and neonatal outcomes.

**What is the impact on readiness and force health protection?**

The collection and reporting of disaggregated racial and ethnic data is crucial to promote understanding of populations of multiracial, Native, and national origins serving in the U.S. military. System improvements in access to and quality of Military Health System obstetric care are needed to address persistent racial disparities and improve force readiness.

Racial and ethnic disparities in adverse obstetric and neonatal outcomes have been widely reported in the U.S. literature. ^1 - 4^ Despite concerted efforts to document and attend to disparities, traditional approaches to racial and ethnic classification often obscure important differences within major racial or ethnic groups (e.g., among diverse Asian and Latino populations) and among multiracial populations, resulting in a bias of averages. ^5^ Additionally, classification methods that restrict racial and ethnic group counts to individuals identifying as single-race and non-Hispanic or Latino lead to significant suppression of American Indian or Alaska Native (AIAN) and Native Hawaiian or Pacific Islander (NHPI) populations: just 23.3% and 39.2% of their national populations, respectively, identified as single-race and non-Hispanic or Latino in the 2020 U.S. Census. ^6^

Assessments of racial and ethnic health disparities in the Military Health System (MHS) are limited by many of the aforementioned data concerns. ^7 - 13^ A more holistic assessment is crucial given the diversity of the population: in 2022, 26.8% of U.S. military service members identified with a historically racialized group (i.e., AIAN, Asian, Black or African American, NHPI, or multiracial), and 17.3% identified as Hispanic or Latino. ^14^ The present study used 1) self-reported racial and ethnic data from personnel records and 2) population-level health care claims data to assess the prevalence of obstetric and neonatal outcomes among U.S. service members by disaggregated race and ethnicity. Additionally, prevalence estimates were calculated for each racial and ethnic group using 2 distinct methods of classification: a mutually exclusive and non-mutually exclusive approach.

## Methods

### Study population

The study population was derived from the U.S. Department of War Birth and Infant Health Research (BIHR) program. The BIHR program is an ongoing surveillance and research effort that identifies live births among military families and captures information on associated pregnancy and infant health outcomes. ^15^ BIHR data comprise military demographic and personnel data from the Defense Manpower Data Center (DMDC) and administrative medical encounter data from the MHS Data Repository. The data repository includes records for all care paid for by TRICARE, the health care plan for service members, retirees, and their families. Covered care spans medical services received at military and civilian facilities within the U.S. and abroad and is available at no cost to active duty service members and their families.

BIHR data were used to identify all live births occurring from January 2010 through December 2021 among pregnant U.S. military service members. Same-sex multiples were excluded due to difficulties distinguishing their neonatal medical records. The study was approved by the Naval Health Research Center Institutional Review Board (protocol NHRC.1999.0003); informed consent was waived in accordance with criteria set forth by Title 32, Code of Federal Regulations, Part 219.

### Measures

Self-reported race and ethnicity data were ascertained from DMDC military personnel records. Values from both the race and ethnicity data fields were considered when assigning race and ethnicity **(Supplementary Table 1)** . The Army and Army Reserve do not allow service members to select multiple categories of race: Multiracial individuals must select a single racial group or “other”. Additionally, all service members can report only 1 ethnicity.

**SUPPLEMENTARY TABLE 1.** Race and Ethnicity Values Reported to Defense Manpower Data Center

	Race	Ethnicity	
Value	Description	Value	Description
1	American Indian or Alaska Native	AA	Asian Indian
2	Asian	AB	Chinese
3	Black or African American	AC	Filipino
4	Native Hawaiian or Other Pacific Islander	AD	Guamanian
5	White	AF	Japanese
100	American Indian or Alaska Native, Asian	AG	Korean
101	American Indian or Alaska Native, Asian, Black or African American	AI	Vietnamese
102	American Indian or Alaska Native, Asian, Black or African American, Native Hawaiian or Other Pacific Islander	AJ	Other Asian descent
103	American Indian or Alaska Native, Asian, Black or African American, Native Hawaiian or Other Pacific Islander, White	AK	Mexican
104	American Indian or Alaska Native, Asian, Black or African American, White	AL	Puerto Rican
105	American Indian or Alaska Native, Asian, Native Hawaiian or Other Pacific Islander	AM	Cuban
106	American Indian or Alaska Native, Asian, Native Hawaiian or Other Pacific Islander, White	AN	Latin American with Hispanic descent
107	American Indian or Alaska Native, Asian, White	AO	Other Hispanic descent
108	American Indian or Alaska Native, Black or African American	AP	Aleut
109	American Indian or Alaska Native, Black or African American, Native Hawaiian or Other Pacific Islander	AQ	Eskimo
110	American Indian or Alaska Native, Black or African American, Native Hawaiian or Other Pacific Islander, White	AR	U.S. or Canadian Indian tribes
111	American Indian or Alaska Native, Black or African American, White	AS	Melanesian
112	American Indian or Alaska Native, Native Hawaiian or Other Pacific Islander	AT	Micronesian
113	American Indian or Alaska Native, Native Hawaiian or Other Pacific Islander, White	AU	Polynesian
114	American Indian or Alaska Native, White	AV	Other Pacific Island descent
115	Asian, Black or African American	BG	Other
116	Asian, Black or African American, Native Hawaiian or Other Pacific Islander	BH	None
117	Asian, Black or African American, Native Hawaiian or Other Pacific Islander, White	ZZ	Unknown
118	Asian, Black or African American, White		
119	Asian, Native Hawaiian or Other Pacific Islander		
120	Asian, Native Hawaiian or Other Pacific Islander, White		
121	Asian, White		
122	Black or African American, Native Hawaiian or Other Pacific Islander		
123	Black or African American, Native Hawaiian or Other Pacific Islander, White		
124	Black or African American, White		
125	Native Hawaiian or Other Pacific Islander, White		
999	Other, Declined to respond, Identification pending		

Data were categorized using 2 distinct approaches: 1) a mutually exclusive (‘alone’) and 2) non-mutually exclusive (‘alone or in combination’) approach. The mutually exclusive (‘alone’) approach first identified Hispanic or Latino individuals, and subsequently grouped non-Hispanic individuals into 1 of the following racial categories: AIAN, Asian, Black or African American, NHPI, multiracial, or unknown. If service members selected multiple categories, they were classified as multiracial. The non-mutually exclusive approach identified all individuals identifying with each group, whether alone or in combination with any other group (i.e., including people who would otherwise be classified as multiracial or Hispanic or Latino). For example, if an individual's self-reported race was “Black or African American” and ethnicity was “Korean,” that individual was categorized as multiracial using the mutually exclusive (‘alone’) approach, and Black or African American, Asian, and Korean using the non-mutually exclusive (‘alone or in combination’) approach.

Risk factors (e.g., age) and indicators of socio-economic disadvantage (e.g., educational attainment, military rank) were identified and treated dichotomously: age at delivery (18-19 years vs. >20 years; <35 years vs. >35 years), educational attainment (bachelor's degree or higher vs. less education), and military rank (officer vs. enlisted).

Three obstetric outcomes were ascertained using International Classification of Diseases, 9th and 10th Revisions (ICD-9/ICD-10), diagnosis codes: cesarean delivery, gestational hypertension, and gestational diabetes **(Supplementary Table 2)** . Cesarean deliveries required notation on either the delivery record or the infant birth record. Gestational hypertension cases required record of associated codes on 1 inpatient or 2 outpatient encounters from 20 weeks estimated gestational age (EGA) to 6 weeks postpartum. Gestational diabetes cases required record of associated codes on 1 inpatient or 2 outpatient encounters from 28 weeks EGA to date of delivery. Cases of pre-existing hypertension and pre-existing diabetes in pregnancy or the year prior to pregnancy were excluded from gestational case definitions. Two neonatal outcomes were also ascertained using ICD-9/ICD-10 diagnosis codes in the infant medical record: pre-term birth (<37 weeks EGA) and low birth weight (<2,500 grams).

**SUPPLEMENTARY TABLE 2.** Case Definitions for Neonatal and Obstetric Outcomes Obtained from Administrative Medical Encounter Data

Variable	Codes	Case Definition
Cesarean delivery	**ICD-9:** V[30-37,39].01, 649.8x, 669.7x, 763.4, 74.[0,1,2,4,99]	Any diagnosis or procedure code on a delivery record or any diagnosis code on an infant birth record	
	**ICD-10:** Z38.[01,31,62,64,66,69], O82.x, P03.4x, O75.82, 10D00Z[0,1,2]		
Gestational hypertension	**ICD-9:** 642.[3,4,5,6]x	One inpatient encounter or 2 outpatient encounters, on different days, from 20 weeks' gestation through 6 weeks postpartum (excluding pre-existing hypertension cases, see below)
	**ICD-10:** O13.x-O15.x	
Pre-existing hypertension	**ICD-9:** 401.x-405.x	Any diagnosis during the year prior to pregnancy or during pregnancy up to 20 weeks gestation
	**ICD-10:** I10.x-I13.x, I15.x	
	**ICD-9:** 642.[0,1,2]x	Any diagnosis during pregnancy up to 20 weeks gestation
	**ICD-10:** O10.x	
Gestational diabetes	**ICD-9:** 648.8x	One inpatient encounter or 2 outpatient encounters, on different days, from 24 weeks gestation through date of delivery (excluding pre-existing diabetes cases, see below)
	**ICD-10:** O24.4x	
Pre-existing diabetes	**ICD-9:** 249.x, 250.x	Any diagnosis during year prior to pregnancy or during pregnancy up to 24 weeks gestation
	**ICD-10:** E08.x, E09.x, E10.x, E11.x, E13.x	
	**ICD-9:** 648.0x	Any diagnosis during pregnancy up to 24 weeks gestation
	**ICD-10:** O24.[0,1,3,8,9]x	
Pre-term birth	**ICD-9:** 765.[0,1], 765.2[1-8], 766.2[1,2], 765.29, 645.[1,2], 644.2[0,1]	Any diagnosis during the delivery hospitalization or in the first 28 days of the infant's life
	**ICD-10:** P07.2[0-6], P07.3[0-9], P08.[21,22], Z3A.[18-42,49], O48.[0,1], O60.1[0,3,4]x[0-9], O60.2[0,2,3]x[0-9], O60.12	
Low birth weight	**ICD-9:** 764.xx, 765.xx, 766.[0,1]	Earliest assigned diagnosis during infant's first 28 days of life; if 2 or more codes assigned, lowest weight prioritized
	**ICD-10:** P05.xx, P07.xx, P08.[0,1]	

### Analysis

The proportion of live births among pregnant U.S. service members was calculated for each racial and ethnic group, alone and alone or in combination with any other group, overall and stratified by age, educational attainment, and military rank. Estimates were presented in the style of a heat map, with color gradients from dark green (indicating lowest risk or disadvantage) to dark yellow (indicating greatest risk or disadvantage). The prevalence of each outcome, as well as 95% confidence intervals (CIs), were calculated for each racial and ethnic group, alone and alone or in combination with any other group. Prevalence was not reported when the numerator included less than 11 cases. Secondary analyses examined prevalence among specific, mutually exclusive racial and ethnic identity intersections (e.g., AIAN *and* White). Data management and statistical analyses were performed using SAS Enterprise Guide, version 7.1 (SAS Institute Inc., Cary, NC).

## Results

The BIHR program captured 1,353,602 live births among U.S. military families from 2010 through 2021, of which 235,608 occurred to pregnant military service members. Analysis of race and ethnicity as an exclusive classification demonstrated births to White ‘alone’ pregnant service members comprised the plurality (47.7%), followed by Black or African American ‘alone’ (22.6%), Hispanic or Latino (16.0%), multiracial (4.1%), Asian ‘alone’ (3.7%), NHPI ‘alone’ (1.4%), and AIAN ‘alone’ (1.3%) **(Table 1)** . When using a non-mutually exclusive racial and ethnic classification approach, the AIAN birth population increased by 209.7% (from 2,985 to 9,245) and the NHPI group increased by 94.0% (from 3,309 to 6,421).

**TABLE 1.** Births to Pregnant Military Service Members by Disaggregated Race and Ethnicity, U.S. Department of Defense Birth and Infant Health Research Program, 2010–2021 (n=235,608)

	Exclusive Classification of Race and Ethnicity		Non-Exclusive Classification of Race and Ethnicity ^a^		
Race and Ethnicity	No.	%	No.	%	Percentage Increase ^b^
American Indian or Alaska Native	2,985	1.3	9,245	3.9	209.7
Asian	8,666	3.7	13,263	5.6	53.0
Asian Indian	210	0.1	431	0.2	105.2
Chinese	525	0.2	558	0.2	6.3
Filipino	2,165	0.9	2,694	1.1	24.4
Japanese	150	0.1	238	0.1	58.7
Korean	591	0.3	674	0.3	14.0
Vietnamese	291	0.1	322	0.1	10.7
Other Asian descent	4,734	2.0	8,346	3.5	76.3
Black or African American	53,226	22.6	60,913	25.9	14.4
Hispanic or Latino	—	—	37,751	16.0	—
Mexican	—	—	9,773	4.1	—
Puerto Rican	—	—	3,046	1.3	—
Cuban	—	—	336	0.1	—
Other Hispanic or Latino descent	—	—	24,596	10.4	—
Native Hawaiian or Pacific Islander	3,309	1.4	6,421	2.7	94.0
Chamorro or Guamanian	159	0.1	185	0.1	16.4
Other Native Hawaiian or Pacific Islander descent	3,150	1.3	6,236	2.6	98.0
White	112,361	47.7	144,266	61.2	28.4
Multiracial	9,619	4.1	—	—	—
Unknown	7,691	3.3	7,691	3.3	—

Abbreviation: No., number

a Non-exclusive classification by race and ethnicity creates a unique count of births within each distinct grouping; thus, birth totals for each race and ethnicity grouping do not sum to total number of births, and percentages do not sum to 100.

b Represents percentage increase from each racial and ethnic group alone to each racial and ethnic group alone or in combination.

Service members identifying as AIAN, Black or African American, and NHPI (both alone and alone or in combination) had higher proportions of pregnant service members younger than age 20 years at delivery and lower proportions of those who completed a bachelor's degree and of officer rank in relation to other groups **(Table 2)** . Patterns were variable among Asian and Latino ethnic groups. Pregnant Filipino service members had lower proportions of college graduates and officers compared with other Asian ethnic groups. Mexican service members demonstrated higher proportions of pregnant service members younger than age 20 years at delivery and lower proportions of those with college education and of officer rank in relation to Cuban and Puerto Rican service members.

**TABLE 2.** Proportion (%) of Pregnant Military Service Members with Selected Characteristics by Disaggregated Race and Ethnicity, Department of Defense Birth and Infant Health Research Program, 2010–2021 (n=235,608) ^a^

	Exclusive Classification of Race and Ethnicity				Non-Exclusive Classification of Race and Ethnicity			
Race and Ethnicity	Age <20 Years	Age >35 Years	College Education or Higher	Sponsor Officer Rank	Age 20 Years	Age >35 Years	College Education or Higher	Sponsor Officer Rank
American Indian or Alaska Native	1.9	6.8	10.8	7.4	1.7	5.2	11.6	8.0
Asian	0.8	14.3	36.7	25.9	0.9	12.7	31.8	23.3
Asian Indian	1.4	15.7	47.1	26.2	1.9	9.7	27.6	15.5
Chinese	0.4	18.5	49.0	25.0	0.4	19.2	48.7	26.3
Filipino	1.0	13.5	28.2	17.2	1.0	12.5	26.2	17.2
Japanese	0.0	13.3	30.7	28.0	0.4	13.4	29.8	30.7
Korean	0.5	19.0	44.0	38.6	0.4	18.4	43.5	39.3
Vietnamese	0.7	16.2	34.7	29.6	0.6	16.5	35.1	30.7
Other Asian descent	0.8	13.5	38.2	28.2	1.0	11.8	31.7	23.7
Black or African American	1.6	8.8	16.1	7.9	1.6	8.4	15.9	7.9
Hispanic or Latino	—	—	—	—	2.1	6.8	13.3	7.9
Mexican	—	—	—	—	3.2	5.4	7.4	4.4
Puerto Rican	—	—	—	—	2.0	8.8	17.5	8.3
Cuban	—	—	—	—	2.7	8.9	20.5	16.4
Other Hispanic or Latino descent	—	—	—	—	1.7	7.1	15.1	9.1
Native Hawaiian or Pacific Islander	1.8	6.4	12.4	7.9	1.4	7.3	15.7	11.0
Chamorro or Guamanian	1.3	10.1	10.1	7.5	2.2	10.8	9.7	8.1
Other Native Hawaiian or Pacific Islander descent	1.8	6.3	12.5	7.9	1.4	7.2	15.8	11.1
White	1.3	9.4	27.6	23.2	1.5	8.5	24.3	19.8
Multiracial	1.4	6.3	17.1	13.5	—	—	—	—
Unknown	1.0	20.3	22.4	41.4	1.0	20.3	22.4	41.4

a Color gradient ranges from dark green (indicating lowest risk or disadvantage among estimates) to dark yellow (indicating greatest risk or disadvantage among estimates).

**FIGURE 1.**
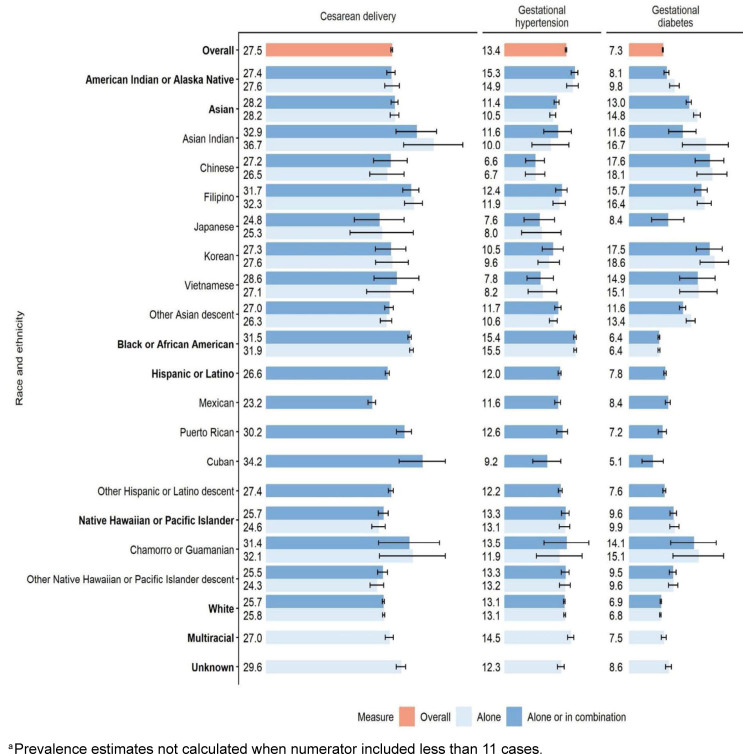
Prevalence (per 100 live births) and 95% Confidence Intervals for Obstetric Outcomes Among Pregnant U.S. Service Members by Disaggregated Race and Ethnicity, Department of Defense Birth and Infant Health Research Program, 2010–2021 ^a^

The overall prevalence of cesarean delivery, gestational hypertension, and gestational diabetes among all live births was 27.5% (95% CI 27.4, 27.7), 13.4% (95% CI 13.3, 13.6), and 7.3% (95% CI 7.2, 7.4), respectively **(Figure 1)** . As a mutually exclusive group, Black or African American service members had a high prevalence of cesarean delivery (31.9%; 95% CI 31.5, 32.3) and gestational hypertension (15.5%; 95% CI 15.2, 15.8), but a low prevalence of gestational diabetes (6.4%; 95% CI 6.2, 6.7). The prevalence of each outcome varied across Asian alone ethnic groups, but skewed below the overall estimate for gestational hypertension, ranging from 6.7% (95% CI 4.5, 8.8) among Chinese service members to 11.9% (95% CI 10.6, 13.3) among Filipino service members. For gestational diabetes, the prevalence among Asian ‘alone’ ethnic groups skewed above the overall estimate, ranging from 13.4% (95% CI 12.5, 14.4) for the ‘other’ Asian descent population to 18.6% (95% CI 15.5, 21.8) for Korean service members. Among Hispanic and Latinos, Puerto Rican and Cuban service members had high prevalences of cesarean delivery (30.2%; 95% CI 28.6, 31.9 and 34.2%; 95% CI 29.2, 39.3, respectively) compared with the overall prevalence and that among Mexican service members (23.2%; 95% CI 22.3, 24.0); however, gestational diabetes was less prevalent among Cuban service members (5.1%, 95% CI 2.7, 7.4) than Mexican service members (8.4%; 95% CI 7.9, 9.4). Gestational diabetes was higher among AIAN alone service members (9.8%; 95% CI 8.8, 10.9) compared to the population inclusive of multiracial and Hispanic or Latino individuals (8.1%; 95% CI 7.5, 8.7). The prevalence of all obstetric outcomes was low among White service members.

**FIGURE 2.**
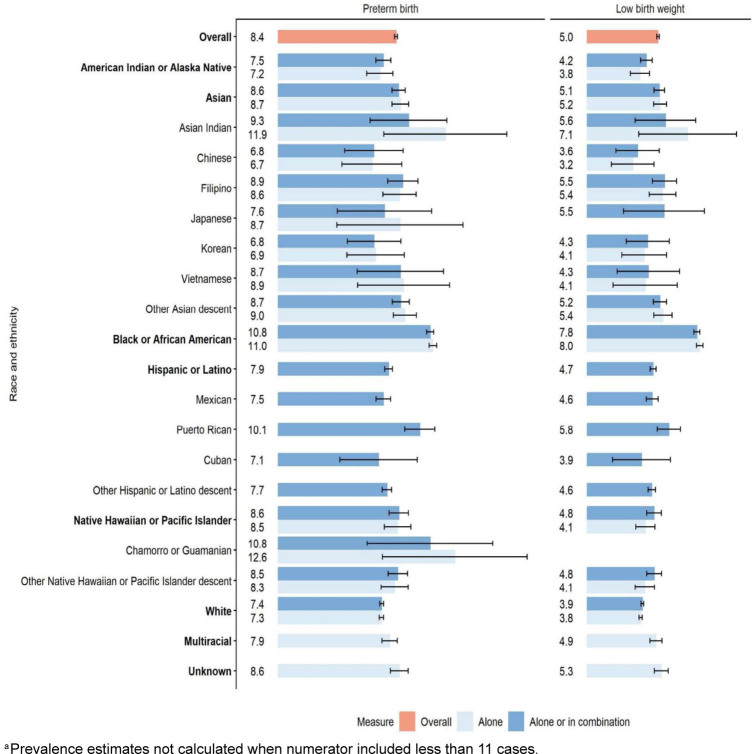
Prevalence (per 100 live births) and 95% Confidence Intervals for Neonatal Outcomes Among Pregnant U.S. Service Members by Disaggregated Race and Ethnicity, Department of Defense Birth and Infant Health Research Program, 2010–2021 ^a^

Overall prevalence of pre-term birth and low birth weight was 8.4% (95% CI 8.3, 8.5) and 5.0% (95% CI 4.9, 5.1), respectively **(Figure 2)** . Black or African American service members had higher prevalences of pre-term birth (alone 11.0%; 95% CI 10.7, 11.3) and low birth weight (alone 8.0%; 95% CI 7.8, 8.2) relative to several other racial and ethnic groups. Prevalence estimates among Asian ‘alone’ and Hispanic or Latino ethnic groups revealed wide variations among both neonatal outcomes, although corresponding CIs were widened for some groups, due to smaller sample sizes. For example, pre-term birth ranged from 6.7% (95% CI 4.5, 8.8) among Chinese service members to 11.9% (95% CI 7.5, 16.3) among Asian Indian service members, and low birth weight ranged from 3.2 (95% CI 1.7, 4.8) among Chinese service members to 7.1 (95% CI 3.7, 10.6) among Asian Indian service members. Hispanic or Latino service members had lower prevalences of pre-term birth (7.9%; 95% CI 7.6, 8.1) and low birth weight (4.7%; 95% CI 4.5, 4.9) than the overall estimate, but prevalence was elevated among Puerto Rican service members (pre-term birth 10.1%; 95% CI 9.0, 11.1; low birth weight 5.8%; 95% CI 5.0, 6.6). The inclusion of multiracial and Hispanic or Latino individuals in estimates for AIAN and NHPI groups, as is reflected in the non-mutually exclusive groupings, resulted in a disproportionate increase in cases of adverse neonatal outcomes, although CIs overlapped.

Outcome prevalences also differed at racial and ethnic identity intersections **(Table 3)** . Black or African American ‘alone’, Black or African American and other Hispanic descent, Filipino, and White and Puerto Rican service members had higher estimates of several adverse outcomes. In contrast, service members who identified as White ‘alone’, White and other Hispanic descent, White and Mexican, and Other Hispanic descent ‘alone’ frequently had lower estimates. Despite similar population sizes, there were also differences in the prevalences of cesarean delivery and gestational diabetes for AIAN ‘alone’ versus AIAN and White service members.

**TABLE 3.** Prevalence (per 100 Live Births) and 95% Confidence Intervals for Neonatal and Obstetric Outcomes by Specific Racial and Ethnic Intersections, Department of Defense Birth and Infant Health Research Program, 2010–2021 ^a^

	Population		Cesarean Delivery			Gestational Hypertension			Gestational Diabetes		
Race and Ethnicity	No.	%	Prevalence	95% CI Lower Limit	95% CI Upper Limit	Prevalence	95% CI Lower Limit	95% CI Upper Limit	Prevalence	95% CI Lower Limit	95% CI Upper Limit
White	112,361	47.7	25.8	25.5	26.0	13.1	12.9	13.3	6.8	6.6	6.9
Black or African American	53,226	22.6	31.9	31.5	32.3	15.5	15.2	15.8	6.4	6.2	6.7
White and other Hispanic descent	15,092	6.4	25.8	25.1	26.5	12.3	11.7	12.8	7.3	6.9	7.7
Unknown ^b^	7,691	3.3	29.6	28.5	30.6	12.3	11.6	13.0	8.6	7.9	9.2
White and Mexican	7,225	3.1	22.6	21.7	23.6	12.2	11.4	12.9	8.1	7.5	8.7
Other Hispanic descent ^c^	4,377	1.9	31.5	30.1	32.9	9.6	8.7	10.4	9.0	8.1	9.8
Asian ^d^	3,683	1.6	27.3	25.9	28.8	11.3	10.3	12.4	13.9	12.8	15.0
Native Hawaiian or Pacific Islander ^d^	3,150	1.3	24.3	22.8	25.8	13.2	12.0	14.4	9.6	8.6	10.6
American Indian or Alaska Native	2,985	1.3	27.6	26.0	29.2	14.9	13.6	16.2	9.8	8.8	10.9
American Indian or Alaska Native and White	2,824	1.2	25.1	23.5	26.7	15.3	13.9	16.6	7.6	6.6	8.6
Black or African American and other Hispanic descent	2,211	0.9	29.0	27.1	30.9	14.4	13.0	15.9	6.5	5.4	7.5
Filipino	2,165	0.9	32.3	30.4	34.3	11.9	10.6	13.3	16.4	14.8	18.0
Mexican ^c^	2,042	0.9	24.5	22.6	26.4	9.1	7.8	10.3	9.8	8.6	11.1
White and Puerto Rican	1,647	0.7	29.1	26.9	31.3	12.1	10.6	13.7	6.0	4.9	7.2
Black or African American and White	1,331	0.6	26.4	24.1	28.8	14.7	12.8	16.6	4.6	3.5	5.0

	Population		Pre-Term Birth			Low Birth Weight		
Race and Ethnicity	No.	%	Prevalence	95% CI Lower Limit	95% CI Upper Limit	Prevalence	95% CI Lower Limit	95% CI Upper Limit
White	112,361	47.7	7.3	7.2	7.5	3.8	3.7	3.9
Black or African American	53,226	22.6	11.0	10.7	11.3	8.0	7.8	8.2
White and other Hispanic descent	15,092	6.4	7.2	6.8	7.6	4.2	3.8	4.5
Unknown ^b^	7,691	3.3	8.6	8.0	9.2	5.3	4.8	5.8
White and Mexican	7,225	3.1	7.6	7.0	8.2	4.7	4.2	5.2
Other Hispanic descent ^c^	4,377	1.9	8.0	7.2	8.8	4.5	3.9	5.1
Asian ^d^	3,683	1.6	9.2	8.3	10.2	5.6	4.9	6.3
Native Hawaiian or Pacific Islander ^d^	3,150	1.3	8.3	7.3	9.2	4.1	3.4	4.8
American Indian or Alaska Native	2,985	1.3	7.2	6.3	8.2	3.8	3.1	4.4
American Indian or Alaska Native and White	2,824	1.2	6.9	6.0	7.9	3.8	3.1	4.5
Black or African American and other Hispanic descent	2,211	0.9	10.4	9.1	11.6	7.6	6.5	8.8
Filipino	2,165	0.9	8.6	7.5	9.8	5.4	4.4	6.3
Mexican ^c^	2,042	0.9	7.8	6.6	8.9	4.6	3.7	5.5
White and Puerto Rican	1,647	0.7	9.0	7.6	10.4	5.4	4.3	6.5
Black or African American and White	1,331	0.6	8.2	6.7	9.7	5.0	3.9	6.2

Abbreviations: No., number.

a Color gradient ranges from dark green (indicating lowest risk or disadvantage among estimates) to dark yellow (indicating greatest risk or disadvantage among estimates). Racial and ethnic identity intersections comprising less than 0.5% of the population (<1,178) were omitted for brevity.

b No specified race or ethnicity.

c No specified race.

d No specified ethnicity.

## Discussion

This study reported the prevalence of selected obstetric and neonatal outcomes among U.S. service members by disaggregated race and ethnicity, revealing varying prevalence within and across racial and ethnic groups. Furthermore, we identified differences between distinct multiracial groups and by using mutually exclusive versus non-mutually exclusive classification structures. These differences are especially important for AIAN and NHPI service members, who are very likely to additionally identify as another race or ethnicity.

We add to a limited body of racial health disparities research conducted among pregnant U.S. military service members. The prevalence of pre-term birth among Black or African American service members was lower than that previously reported using 2003-2014 data (11.0% vs. 11.5%), while low birth weight was more prevalent in the present study (8.0% vs. 7.7%). ^15^ Prevalence of both neonatal outcomes, as well as of cesarean delivery and gestational hypertension, remained higher among Black or African American service members compared with all other major racial and ethnic groups. These findings underscore the continued relevance of disparities previously identified for neonatal mortality, severe maternal morbidity, and pregnancy-related mortality, and counter suggestions that comprehensive health coverage alone eliminates health disparities. ^8 - 10 , 16^

Disaggregation of the population identifying as Asian or Pacific Islander elucidated marked differences for each group overall and across specific ethnic groups. For low birth weight, overall estimates were 4.1% among NHPI alone service members and 5.2% among Asian alone service members, whereas the aggregated estimate using data from 2003-2014 was 4.9%. ^15^ Differences were also pronounced for gestational diabetes, with Asian alone service members having a 49.5% increased risk compared with NHPI alone service members. Findings parallel prior work documenting higher risk for gestational diabetes among Asian populations compared with other major racial and ethnic groups, as well as uniquely high risk among Asian Indian, Vietnamese, and Filipino ethnic groups. ^17 , 18^ We also noted prevalence estimates for Chinese and Korean service members that were higher than typically reported ^17 , 18^ ; this finding may reflect differences between civilian and active duty populations related to place of birth, childhood socio-economic status, education, and age at delivery. Observed differences among Asian ethnic groups provide further justification for acknowledgment of aggregation as a potential fallacy. ^5^

More inclusive, non-mutually exclusive definitions of race and ethnicity proved particularly effective for capturing births among AIAN and NHPI service members, as the numbers of births attributed to these populations increased by 209.7% and 94.0%, respectively, if compared to a mutually exclusive approach. Although meaningful differences in estimates between mutually exclusive and non-mutually exclusive groups were difficult to ascertain due to wide CIs, with the exception of gestational diabetes, point estimates were consistently higher among the NHPI ‘alone or in combination’ population, indicating greater risk for multiracial NHPI populations. For AIAN service members, gestational diabetes was higher among those identifying as AIAN alone. Prior work has shown the AIAN ‘alone’ population experienced increased economic disadvantage and decreased life expectancy relative to the multiracial AIAN population, ^19 , 20^ whereas the multiracial AIAN population experienced increased depression and mental distress. ^21^ Our findings of disparate estimates by multiracial identity, therefore, contribute further nuance to awareness of Native health in the U.S.

There are some notable limitations with military personnel race and ethnicity data that affected this work. First, in the Services overall, only 1 ethnic identity could be reported; and in the Army and Army Reserve (which accounted for nearly 40% of all births in this cohort), soldiers could not report identity with multiple racial groups. Consequently, the multi-racial population was under-estimated. Second, although self-reported, these records remain subject to data entry and administrative errors. Finally, detailed ethnicity is available only for Asian and Hispanic or Latino groups, hampering understanding of diversity among White, Black or African American, and NHPI service members.

The approach to this work, through the presentation of estimates for mutually exclusive and non-mutually exclusive racial and ethnic groups, as well as specific groups comprising a significant proportion of the population, mirrors recommendations in the 2024 Office of Management and Budget guidance for the maintenance, collection, and presentation of racial and ethnic data. ^22^ Our findings underscore that a singular, mutually-exclusive approach to racial and ethnic classification is insufficient for understanding racial health disparities: It disproportionately obscures AIAN and NHPI populations and homogenizes multiracial populations. ^23 , 24^ As the U.S. population is increasingly multiracial, ^25^ disaggregation will only grow more pertinent. Ultimately, while application of multiple approaches to racial and ethnic classification may not always be feasible, researchers should consider the implicit biases or assumptions reflected in their selected approach. ^26^ Greater attention to the collection and reporting of disaggregated racial and ethnic health data will improve understanding of health outcomes within and beyond the MHS. ^13^
